# Functional Anatomy of Recognition of Chinese Multi-Character Words: Convergent Evidence from Effects of Transposable Nonwords, Lexicality, and Word Frequency

**DOI:** 10.1371/journal.pone.0149583

**Published:** 2016-02-22

**Authors:** Nan Lin, Xi Yu, Ying Zhao, Mingxia Zhang

**Affiliations:** 1 Key Laboratory of Behavioral Science, Institute of Psychology, Chinese Academy of Sciences, Beijing, 100101, China; 2 Laboratories of Cognitive Neuroscience, Division of Developmental Medicine, Department of Medicine, Boston Children’s Hospital, Boston, MA, 02115, United States of America; 3 Harvard Medical School, Boston, MA, 02115, United States of America; 4 State Key Laboratory of Cognitive Neuroscience and Learning & IDG/McGovern Institute for Brain Research, Beijing Normal University, Beijing 100875, China; University of Leicester, UNITED KINGDOM

## Abstract

This fMRI study aimed to identify the neural mechanisms underlying the recognition of Chinese multi-character words by partialling out the confounding effect of reaction time (RT). For this purpose, a special type of nonword—transposable nonword—was created by reversing the character orders of real words. These nonwords were included in a lexical decision task along with regular (non-transposable) nonwords and real words. Through conjunction analysis on the contrasts of transposable nonwords versus regular nonwords and words versus regular nonwords, the confounding effect of RT was eliminated, and the regions involved in word recognition were reliably identified. The word-frequency effect was also examined in emerged regions to further assess their functional roles in word processing. Results showed significant conjunctional effect and positive word-frequency effect in the bilateral inferior parietal lobules and posterior cingulate cortex, whereas only conjunctional effect was found in the anterior cingulate cortex. The roles of these brain regions in recognition of Chinese multi-character words were discussed.

## Introduction

Word recognition is a basic component of reading and has been studied extensively by different methods. In particular, neuroimaging studies have deepened our understanding of word recognition by providing insights into its functional anatomy [1–3]. The most direct way to explore brain structures associated with word recognition is to compare brain activation evoked by words with that evoked by nonwords. The difference in brain activation elicited by the word and nonword conditions, which is often referred to as the lexicality effect, has been observed in extensive brain regions, indicating that skilful word recognition requires/evokes the interplay of various neural networks and processes [1, 2].

An important limitation of the lexicality effect is that it confounds brain activation specific to lexical processes with that reflecting the domain-general processes sensitive to reaction time (RT). At the behavioral level, nonwords require longer RTs to process than words in lexical decision [2]. At the brain level, the brain regions showing the lexicality effect (e.g., the supplementary motor area, inferior frontal gyrus, precentral sulcus, and angular gyrus) [1, 2, 4] largely overlap with regions whose activation is sensitive to RT across various (linguistic and nonlinguistic, semantic and nonsemantic) tasks and is thought to reflect domain-general processes such as attention, short-term memory, and executive processes [5–9]. The confounding of lexicality and RT effects has led to considerable disagreement on the precise role of the identified brain regions, with some studies interpreting the activation as lexical and semantic processes [1, 4, 10] whereas the others interpreting the activation in terms of domain-general processes [2, 9, 11]. Therefore, new experimental paradigms are needed to dissociate the brain activation reflecting lexical processes from that associated with the RT effect.

In the current study, we focused on a particular kind of words, namely Chinese multi-character words, and explored the functional anatomy of their recognition by partialling out the confounding effect of RT. Chinese words are comprised of one or more characters. Multi-character words constitute the majority of the type frequency counts in corpora (about 80–97% across different corpora) [12–14]. Behavioral studies have demonstrated that lexical representations of multi-character words have strong influences on Chinese reading, which cannot be fully accounted for by character-level processing [15–18]. For instance, the frequencies of multi-character words have been reported to modulate the fixation times on them during text reading [15]. Inserting spaces between words (which is unnatural in Chinese) did not interfere with Chinese text reading, but inserting spaces within multi-character words did [16]. Moreover, the efficiency of character recognition was strongly affected by the existence of word boundaries between them [17].

Although recognition of multi-character words is crucial for Chinese reading, most neuroimaging studies on Chinese language processing have focused on character-level processes [19], leaving the brain mechanisms underlying multi-character word recognition largely unknown. To our knowledge, only two neuroimaging studies have explored the brain activation reflecting Chinese multi-character word recognition by controlling the effects associated with character-level processes [20, 21]. In the first study, Zhang et al. [20] compared the brain activation evoked by multi-character words with that evoked by multi-character nonwords and found extensive brain regions showing the lexicality effect, including the inferior frontal gyrus, middle frontal gyrus, superior frontal gyrus, supplementary motor area, cingulate cortex, precuneus, angular gyrus, middle temporal gyrus, and fusiform gyrus. In the second study, Zhan et al. [21] explored the neural mechanisms underlying Chinese multi-character word recognition by comparing the brain activation associated with mixed and pure pseudohomophones, which were created by replacing one and both constituent characters of two-character words with their homophones, respectively. At the behavioral level, Zhan et al. found that rejection of mixed pseudohomophones requires longer RTs than that of pure pseudohomophones, which is consistent with the findings of a prior behavioral study [22] and indicates that the mixed pseudohomophones activate the corresponding real words to some extent. At the brain level, Zhan et al. found greater activation for mixed pseudohomophones in the left inferior frontal gyrus and left inferior parietal lobule (IPL). The authors interpreted the roles of these emerged brain regions as processing semantic information and integrating phonological and orthographic information during word recognition.

In these two previous studies, the confounding effect of RT was neither controlled nor considered. There was a large RT difference between the word and nonword conditions in the first experiment, and the emerged brain regions largely overlapped with the brain regions associated with the RT effect. In the second study, although the RT difference between the mixed and pure pseudohomophone conditions was much smaller than that between the word and nonword conditions, it was still significant. Therefore, the confounding effect of RT was not partialled out.

In the present study, we dissociated the brain activation reflecting Chinese multi-character word recognition from that associated with the RT effect by jointly examining the brain activation evoked by multi-character words, regular multi-character nonwords, and a special type of multi-character nonwords, namely, transposable nonwords. The transposable nonwords were constructed by transposing the characters of real words. For example, “演表” is a transposable nonword constructed by swapping the constituents of the two-character word “表演” (which means “performance”). Behavioral studies have showed that the representation of a Chinese multi-character word can be activated by the transposition of its characters. Taft, Zhu, and Peng [23] investigated the transposed-character effect by using the standard lexical decision task. They found that the rejection of transposable nonwords requires longer RTs than that of regular nonwords, indicating that participants tended to misperceive the transposable nonwords as their base words. Gu, Li, and Liversedge [24] examined early-stage processing of transposable nonwords by using a masked priming lexical decision paradigm and a gaze-contingent display-change paradigm. In the priming lexical decision task, after a brief exposure (60 ms) to a prime, participants were presented with a target stimulus and were asked to make a lexical decision to the target stimulus. In the gaze-contingent display-change reading task, readers were provided with a parafoveal preview stimulus that then changed to a target word when the participants’ eyes fixated on it. The results showed that the RTs in the lexical decision task and reading times on the target words were shorter in the transposable-nonword condition (in which the prime or the preview was a transposable nonword whose base word was the target word) than in the regular-nonword condition (the prime or the preview was a regular nonword that was unrelated to the target word), indicating that the transposable nonwords can prime their base words from an early stage of word recognition (see another recent study [25] for similar findings on four-character transposable nonwords).

This transposed-character effect can be achieved through two possible processing pathways. Since Chinese is a morphosyllabic language where a character is usually mapped onto one syllabic morpheme, the transposable nonword may activate its base word either through morpheme level processing by employing mechanisms similar to that underlying the transposed-morpheme effect in alphabetic languages (e.g., “walkjay” activating “jaywalk”, “boycow” activating “cowboy”) [26, 27] or through whole-word level processing by employing mechanisms similar to that underlying the transposed-letter effect in alphabetic languages (e.g., “probelm" activating “problem”, “fo” activating “of”) [28–32]. In some early studies, the transposed-character effect in Chinese word reading was thought to reflect morpheme level processing only [33]. However, Gu et al. [24] investigated the recognition of transposable nonwords constructed based on both single- and multiple-morpheme two-character words and found similar transposed-character effects in both the single- and multiple-morpheme conditions. Thus, the transposed-character effect in Chinese reading cannot be explained by morpheme level processing alone.

In order to identify the neural mechanisms underlying Chinese multi-character word recognition without the confounding effect of RT, the common brain activation involved in both the lexicality and transposed-character effects was examined through the conjunction analysis. Conjunction analysis is a classic approach to eliminate the confounding effects in neuroimaging studies [34]. While a single contrast looks for activation differences between one pair of conditions, a conjunction analysis looks for the common activation differences between two or more pairs of conditions. Therefore, a confounding effect can be eliminated through a conjunction analysis if it is not shared, in the same direction, by all contrasts included in the analysis. The use of conjunction analyses makes controlling confounding effects easier, because it is not necessary to control all confounding effects in a single contrast. In other words, the only constraint on setting contrasts for a conjunction analysis is that the effect of interest should be the only effect that is shared by all contrasts.

In the present study, the effect of interest was brain activation associated with accessing and processing of representations of Chinese multi-character words. This effect was shared by the two contrasts of “transposable nonwords > regular nonwords” and “words > regular nonwords” because both words and transposable nonwords can activate the representations of Chinese multi-character words [23]. In contrast, the directions of RT effects associated with the two contrasts are reversed in lexical decision: transposable nonwords are more difficult to process than regular nonwords, whereas real words are easier to process than regular nonwords [23]. In other words, the two contrasts did not share the same RT effect, with the contrast of “transposable nonwords > regular nonwords” associated with a positive RT effect and that of “words > regular nonwords” associated with a negative RT effect. Both directions of RT effects have demonstrated strong confounding effects in neuroimaging studies (for positive RT effect, see [9]; for negative RT effect, see [6]). Through conjunction analysis, the opposite RT effects would be cancelled off and the common effect associated with the two contrasts, i.e., brain activation specific to the processes of word recognition would be identified. Given the strong and extensive RT effects demonstrated by recent fMRI studies [6, 9], we expected that the results of our conjunction analysis would be largely different from those of previous studies that did not control the RT effect [20]. In particular, activation of extensive brain regions in support of domain-general processes, which is sensitive to RT, would be eliminated from our results. Instead, a small subset of the brain regions observed in previous studies, whose functions are specific to accessing and processing the representations of Chinese multi-character words, would be identified.

## Methods

### Ethics statement

The study was approved by the Institutional Review Board of the National Key Laboratory of Cognitive Neuroscience and Learning, Beijing Normal University, and was conducted according to the principles expressed in the Declaration of Helsinki and the guidelines issued by the local ethical committee. Before the experiment, each participant read and signed a written informed consent form issued by the Institutional Review Board of the Beijing Normal University Imaging Center for Brain Research.

### Participants

Eighteen healthy undergraduate and graduate students (16 females) participated in this experiment. The average age of the participants was 21.6 years (SD: 2.7 years). All participants were right handed and native Chinese speakers with no history of psychiatric or neurological disorders.

### Design and materials

A lexical decision paradigm was applied in this study. Three conditions were employed, namely, transposable nonwords (e.g., “演表”), regular nonwords (e.g., “踪安”), and words (e.g., “统治”, which means “rule”). Thirty-five transposable nonwords and 35 regular nonwords were constructed, which were all two characters long. For transposable nonwords, transposing the constitute characters will result in real words (e.g., “演表” is a nonword, but the transposed characters “表演” form a word that means “performance”). For regular nonwords, character transposition would not form real words (e.g., neither “踪安” itself nor the transposition of its characters “安踪” is a real word). One hundred and seventy-five two-character real words, with frequency range from 0.8 to 81.5 per million [35], were also selected to form the real-word condition. Across the three conditions, the stimuli were balanced in terms of character frequency [14] and stroke number [Character frequency (the 1st character): *F* (2, 242) = 1.48, *p* = 0.231; Character frequency (the 2nd character): *F* (2, 242) < 1; Stroke number (the 1st character): *F* (2, 242) = 2.16, *p* = 0.117; Stroke number (the 2nd character): *F* (2, 242) = 0.30, *p* = 0.743; see Table 1 for the *Mean*s and *SD*s]. All words and nonwords were repeated once in the experiment, resulting in 70 transposable nonword trials, 70 regular nonword trials, and 350 word trials. The inclusion of a relatively large number of word stimuli was designed for additional investigation on the fine-grained semantic dimensions of the words, which was unrelated to the interest of the present study. The relative low probability of the nonword items may result in additional cost in decision-making and therefore enlarge the RT difference between the word and regular nonword conditions. However, the trial numbers were well-matched in the contrast between the transposable and regular nonword conditions. Therefore, any confounding effect associated with unequal trial numbers between conditions would be eliminated by the conjunction analysis on the two contrasts and would not affect our final results.

**Table 1:** Mean character frequency and stroke number for words, transposable nonwords and regular nonwords.

	Words		Transposable nonwords		Regular nonword	
	1st character	2nd character	1st character	2nd character	1st character	2nd character
Character frequency	437 (761)	542 (806)	581 (729)	604 (813)	664 (971)	544 (996)
Stroke number	9.6 (2.7)	9.1 (3.0)	8.5 (3.4)	9.4 (2.8)	9.2 (2.9)	9.3 (2.9)

*SD*s are included in parentheses.

### Procedure

An event-related design was applied with five runs, each lasting 5 min 48 s. Every run included 14 transposable nonword trials, 14 regular nonword trials, and 70 word trials. The background was black, and all nonwords and words were white. In each trial, the target word/nonword appeared for 1.5 s, followed by a jittered interval of at least 0.5 s. Participants were instructed to determine whether the present stimulus was a real word or not, and then press a button with their right index or middle finger as quickly and accurately as possible. The order of test trials and the length of jittered intervals were optimized using Optseq software (http://surfer.nmr.mgh.harvard.edu/optseq/). Before the actual experiment, each participant completed a practice run outside the scanner with additional stimuli, and the procedure of the practice run was identical to that of the actual experiment.

### Acquisition and analysis of magnetic resonance imaging data

Magnetic resonance imaging (MRI) data were collected on a 3T Siemens Trio Tim scanner (Beijing Normal University, China). Blood oxygen level-dependent signal data were acquired with a T2*-weighted gradient echo planar imaging sequence (TR: 2000 ms; TE: 30 ms; flip angle: 90°; matrix size: 64 × 64; 33 slices; voxel size: 3.125 mm × 3.125 mm × 4 mm). High-resolution 3D structural data were acquired with a 3DMPRAGE sequence in the sagittal plane (TR: 2530 ms; TE: 3.39 ms; flip angle: 7°; matrix size: 256 × 256; 128 slices; voxel size: 1.33 mm × 1 mm × 1 mm).

MRI data preprocessing was performed using Data Processing Assistant for Resting-State fMRI (DPARSF, http://www.restfmri.net) [36], which is based on SPM8 software (http://www.fil.ion.ucl.ac.uk/spm) and Resting-State fMRI Data Analysis Toolkit (REST, http://www.restfmri.net) [37]. The first five volumes of each run were discarded to allow for steady-state magnetization. The remaining imaging data were reprocessed for slice timing correction, head motion correction, spatial normalization, and spatial smoothing. During normalization, the structural image of each participant was first co-registered to the mean functional image and then segmented. The parameters obtained in the segmentation were used to normalize the functional images of each participant onto the Montreal Neurological Institute (MNI) space. Spatial smoothing was performed with a 6 mm full-width half-maximum Gaussian filter.

Statistical analyses were conducted using a two-level mixed-effects model. At the first level, a general linear model (GLM) was applied to explore the fixed effects within each subject. For the two nonword conditions, the onset of each type of event was modeled using a constant impulse regressor. For the word condition, a variable impulse regressor, with values proportional to the log-transformed word frequencies, was included to model the word-frequency effect in addition to a constant impulse repressor representing the event onsets. All of these constant and variable impulse repressors were convolved with a standard hemodynamic response function and entered into GLM, together with the six head motion parameters as nuisance variables. A high-pass filter with cutoff at 113 s was further employed to remove low-frequency noises, such as physiological noise from cardiac and respiratory cycles. After model estimation, four contrast maps, each representing the beta value for one event type of interest, i.e., transposable nonwords, regular nonwords, real words, and word frequency, were created for each subject.

At the second level, group analysis was performed by entering subject-wise contrast maps into within-subject ANOVA. The effects of transposable nonwords and lexicality were first examined through contrasts of transposable nonword versus regular nonword and word versus regular nonword, respectively. A conjunction analysis was then conducted to explore the brain regions that showed greater activation for both transposable nonwords and real words than that for regular nonwords. For conventional contrasts, threshold was set at *p* < 0.001, k = 52, corresponding to cluster level significance at *α* < 0.05, based on REST AlphaSim (http://www.restfmri.net). For conjunction analysis, a lenient threshold at *p* < 0.001, k = 10 was adopted. The results of the fMRI data analyses were presented using Brainnet Viewer software [38].

Finally, a post hoc analysis of the word-frequency effect was conducted to further explore the functional roles of the emerged regions. Both positive and negative word-frequency effects have been observed in previous neuroimaging studies on word reading. The positive effects of word frequency (i.e., stronger activation for high-frequency words than for low-frequency words) were interpreted as neural regions that supported semantic processing because access to word meaning is more extensive and automatic in the case of high-frequency words. By contrast, the negative effects of word frequency (i.e., stronger activation for low-frequency words than for high-frequency words) were associated with orthographic and/or phonological processes because low—frequency words require more effortful orthographic processes and phonological mediation [3, 39, 40]. Therefore, by testing the word-frequency effect, the cognitive processes associated with the emerged regions can be inferred. To this end, each region obtained from the conjunction analysis was defined as a region of interest (ROI), where the beta values for the frequency effect were extracted and averaged across all the voxels within that ROI for each subject. One-sample t-test was then conducted to determine whether the ROI-specific beta values were significantly higher or lower than zero.

## Results

### Behavioral results

**Fig 1:**
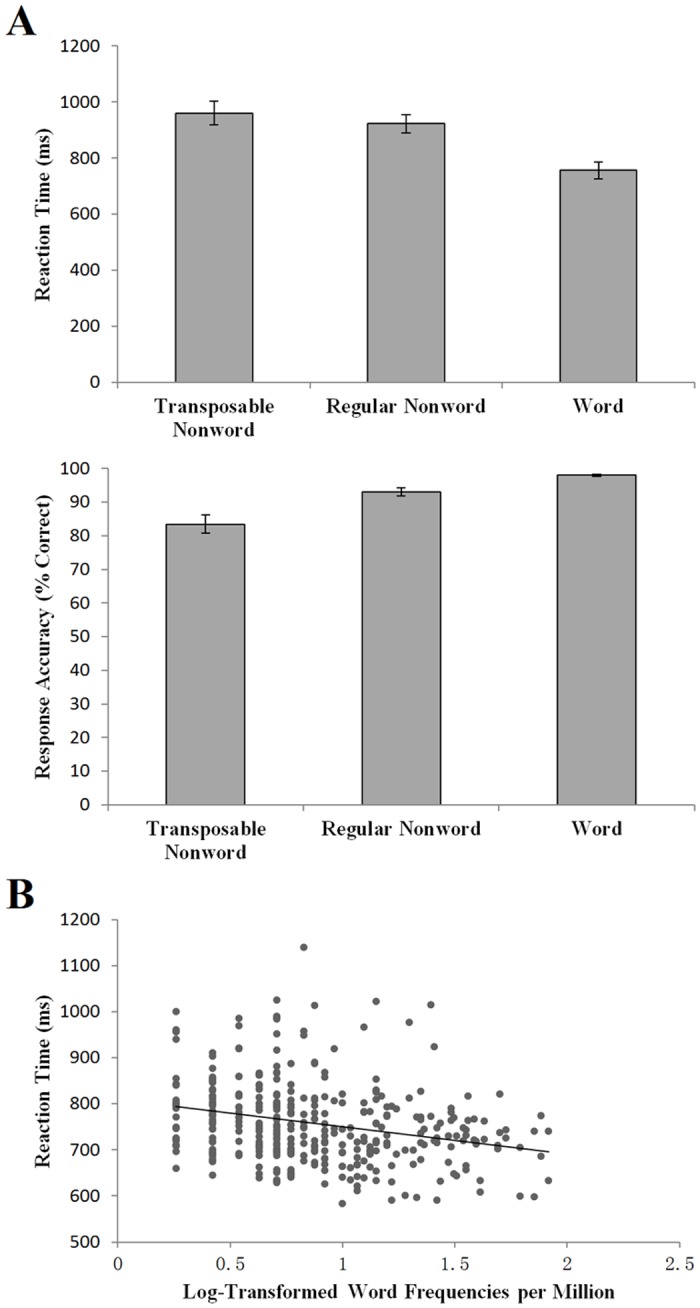
Behavioral results of the fMRI experiment. (A) the reaction times and accuracies of the word, regular nonword, and transposable nonword conditions (the error bars depict the unbiased standard errors of the data); (B) correlation between the log-transformed word frequencies and RTs across items of the word condition (r = -0.26, p < 0.001).

RTs and accuracy data were collected while the participants performed the lexical decision task in the scanner. The mean RTs of the word, regular nonword, and transposable nonword conditions were 756 (*SD*: 129), 922 (*SD*: 135), and 961 (*SD*: 177) ms, respectively (Fig 1A). Transposable nonwords were significantly slower to respond to than regular nonwords [*t* (17) = 2.26, *p* = 0.037], which in turn was significantly slower than real words [*t* (17) = 10.24, *p* < 0.001]. The mean accuracies of the word, regular nonword, and transposable nonword conditions were 98.0% (*SD*: 1.2%), 93.0% (*SD*: 4.8%), and 83.4% (*SD*: 11.4%), respectively (Fig 1A). The word condition presented a significantly higher accuracy rate than the regular nonword condition [*t* (17) = 4.47, *p* < 0.001]. The regular nonword condition presented a significantly higher accuracy rate than the transposable nonword condition [*t* (17) = 3.97, *p* < 0.001]. Consistent with previous studies [23], real words were easier to respond to than nonwords, and transposable nonwords were more difficult to respond to than regular nonwords. In addition, an item-based analysis within the word condition was conducted to examine correlation between the log-transformed word frequencies and RTs (Fig 1B). The results showed a significant word-frequency effect (*r* = -0.26, *p* < 0.001), ensuring the validity of post hoc analysis of the word-frequency effect at the brain level.

### FMRI results

**Fig 2:**
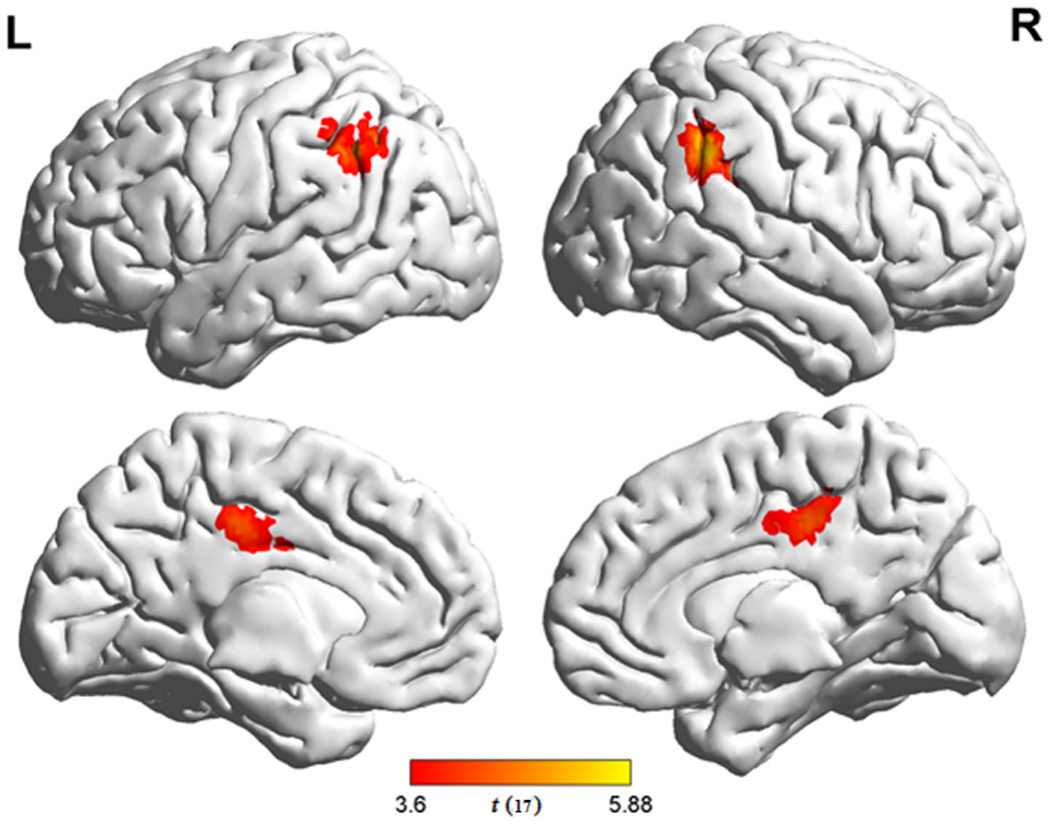
Results of comparisons between transposable and regular nonwords. Brain regions that demonstrated stronger activation to transposable nonwords are shown in warm color. By contrast, no regions showed stronger activation to regular nonwords. False positive rate was set at *α* < 0.05 (*p* < 0.001, k = 52) using REST AlphaSim.

**Fig 3:**
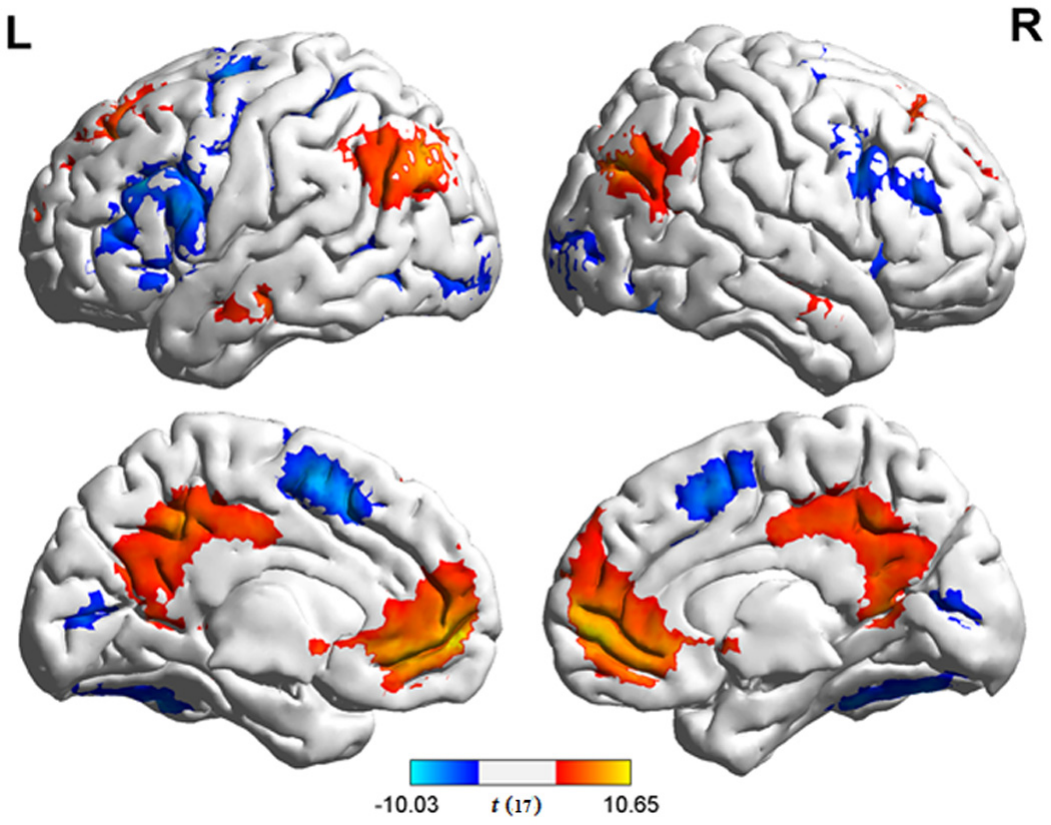
Results of comparisons between words and regular nonwords. Brain regions that demonstrated stronger activation to words are shown in warm color. Brain regions that demonstrated stronger activation to regular nonwords are shown in cold color. False positive rate was set at *α* < 0.05 (*p* < 0.001, k = 52) using REST AlphaSim.

The results of individual contrasts for the effects of transposable nonwords and lexicality are summarized in Table 2. Compared with regular nonwords, transposable nonwords elicited greater activation in the bilateral IPLs (bIPLs) and bilateral posterior cingulate gyri, whereas no brain region survived the significant threshold for the reversed contrast (Fig 2). For the comparison between words and regular nonwords, extensive brain regions showed stronger activation to words, including the medial prefrontal cortex, bilateral superior frontal gyri, bIPLs, bilateral posterior cingulate gyri, bilateral parahippocampal gyri, bilateral middle temporal gyri, and left cerebellum. The opposite contrast revealed greater activation for regular nonwords than for words in the bilateral medial precentral gyri, bilateral middle frontal gyri, bilateral inferior frontal gyri, bilateral superior parietal lobule, bilateral fusiform gyri, bilateral inferior occipital gyri, bilateral middle occipital gyri, bilateral cerebella, and left thalamus (Fig 3).

**Table 2:** Brain regions that showed the effects of transposable nonwords and lexicality.

Contrast	Anatomical region	Cluster size (voxels)	MNI coordinates (x, y, z)			Peak t value
**Transposable nonword > Regular nonword**						
	Right Inferior Parietal Lobule	189	57	-39	27	5.88
	Left Inferior Parietal Lobule	149	-54	-51	36	5.49
	Posterior Cingulate Gyrus	102	6	-30	42	4.59
**Word > Regular nonword**						
	Medial Prefrontal Cortex and Bilateral Superior Frontal Gyrus	2583	0	57	0	10.65
	Left Inferior Parietal Lobule	608	-48	-75	33	8.64
	Right Inferior Parietal Lobule	512	45	-75	33	8.59
	Posterior Cingulate Gyrus	1399	-6	-45	42	7.95
	Left Parahippocampa Gyrus	64	-30	-42	-6	6.48
	Left Middle Temporal Gyrus	129	-63	-15	-18	6.47
	Right Parahippocampa Gyrus	56	30	-33	-9	5.30
	Right Middle Temporal Gyrus	109	66	-15	-12	5.20
	Left Cerebellum	88	-39	-75	-42	4.65
**Regular nonword > Word**						
	Bilateral Medial Precentral Gyri, Left Inferior Frontal Gyrus, Middle Frontal Gyrus, and Superior Parietal Lobule	3502	-9	9	54	10.03
	Bilateral Fusiform Gyri, Inferior Occipital Gyri, Middle Occipital Gyri, and Cerebella	3455	33	-57	-27	8.50
	Right Inferior Frontal Gyrus	805	30	24	3	7.55
	Right Superior Parietal Lobule	194	30	-60	48	6.01
	Right Middle Frontal Gyrus	185	27	3	54	5.65
	Left Thalamus	70	-9	-15	9	5.60

**Fig 4:**
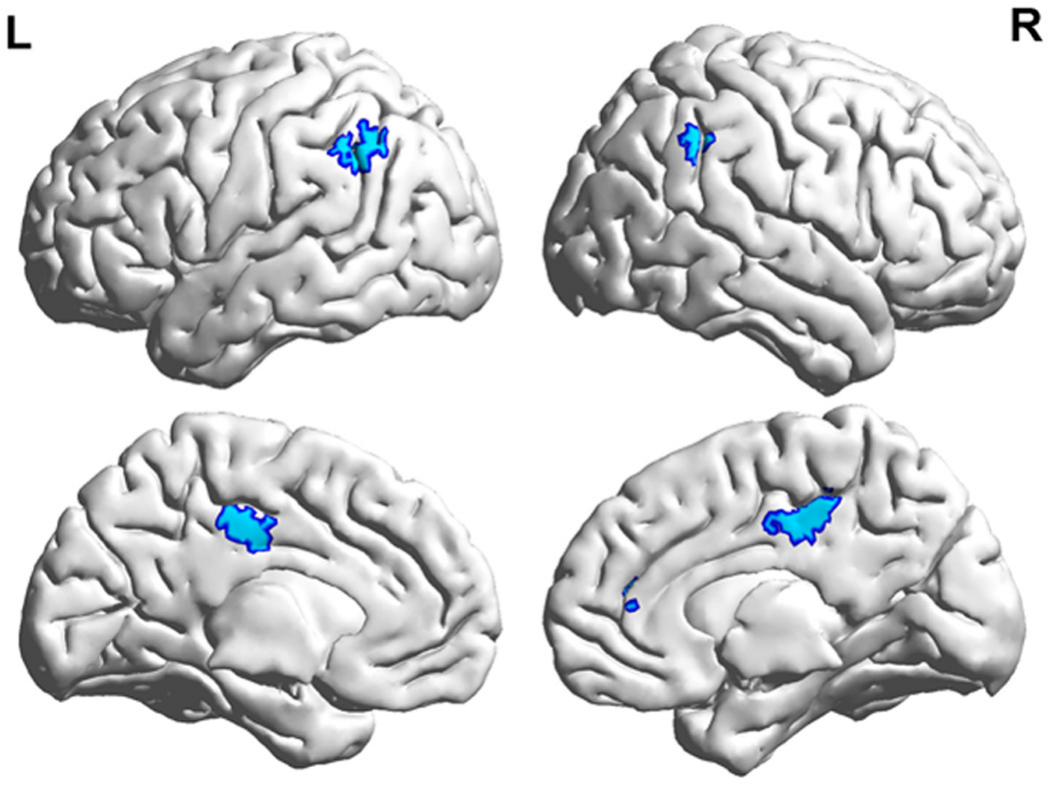
Conjunction results for the effects of transposable nonwords and lexicality (*p* < 0.001 uncorrected, k = 10).

The conjunction of the effects of transposable nonwords (“transposable nonword > regular nonword”) and lexicality (“word > regular nonword”) was observed in the bIPLs (left: 75 voxels; right: 21 voxels), bilateral posterior cingulate gyri (90 voxels), and right anterior cingulate gyrus (24 voxels) (Fig 4). In the ROI analyses of the word-frequency effect, significant positive word-frequency effects were observed in the ROIs located in the right IPL [mean beta value (SD): 0.66 (0.55); *t* (17) = 2.35, *p* < 0.001] and bilateral posterior cingulate gyri [mean beta value (SD): 0.42 (0.61); *t* (17) = 2.91, *p* = 0.010], which survived at the corrected threshold for multiple comparisons (*p* < 0.0125). The left IPL showed a positive word-frequency effect that survived only at the uncorrected threshold [mean beta value (SD): 0.37 (0.68); *t* (17) = 2.35, *p* = 0.031]. No word-frequency effect existed in the right anterior cingulate gyrus [mean beta value (SD): 0.07 (0.45); *t* (17) < 1].

## Discussion

In this study, neural mechanisms underlying recognition of Chinese multi-character words were investigated by examining the brain activation evoked by Chinese multi-character words, transposable nonwords, and regular nonwords. Compared with regular nonwords, transposable nonwords and real words both evoked stronger activation in the bIPLs, bilateral posterior cingulate gyri, and right anterior cingulate gyrus. ROI-based analysis showed that the bIPLs and bilateral posterior cingulate gyri demonstrated a positive word-frequency effect.

Compared to previous studies on Chinese word recognition, a major improvement in the present study was the use of conjunction analysis to exclude the confounding effect of RT. The RT effect modulates activation and deactivation in extensive brain regions across different tasks [6, 9]. In analyses based on a single effect associated with word recognition (e.g., the lexicality effect, the word frequency effect), the effect of interest is often confounded by that of RT [2, 3, 11]. Consistently, current results on the lexicality effect showed activation difference between the word and nonword conditions in extensive brain regions, which overlapped largely with task-related activation and deactivation networks [41], whose activation is modulated by RT in various tasks [6, 9]. Therefore, the lexicality effect may reflect not only brain mechanisms of word recognition but also those of domain-general processes sensitive to RT. To address this issue, we introduced transposable nonwords into the present study. In a lexical decision task, transposable nonwords represent the most difficult condition (which requires the longest RT), followed by regular nonwords, with the real-word condition being the easiest. By examining the conjunctional effect on the two contrasts “transposable nonwords > regular nonwords” and “words > regular nonwords”, the RT effect was disregarded, and the common processes, comprising the access and processing of Chinese multi-character word representations, were identified. Therefore, conjunction analysis of the effects of transposable nonwords and lexicality enhanced the selectivity of the results, making the interpretation more straightforward and reliable.

A further question for interpreting our findings is: what specific roles do the emerged brain regions play in recognition of Chinese multi-character words? Although the present study did not directly dissociate semantic, orthographic, or phonological activation, the word-frequency effect in the emerged regions has provided useful clues about their functional roles. By using ROI analyses, we found that the bIPLs and the posterior cingulate cortex showed greater activation for high-frequency words than for low-frequency words. These findings are consistent with those of previous research on Chinese and English reading [3, 40] and have been interpreted in terms of semantic processing due to access to word meaning being more extensive and automatic in the case of high-frequency words.

Besides the word-frequency effect, previous studies on processing of Chinese and alphabetic languages have also provided a large body of evidence in support of the involvement of the emerged regions in semantic processing. The importance of left IPL for word reading was first mentioned in the 19th century [42] and has been well demonstrated in recent years through the use of modern techniques, such as PET, fMRI, and TMS [3, 43–46]. In early neurological studies, the left IPL was hypothesized as a visual memory center for words [42]. However, recent studies have found that bIPLs, especially the posterior part (bilateral angular gyri), are implicated in accessing and integrating semantic information [3, 10, 47–52]. In a previous fMRI study, Chou et al. [47] investigated the semantic integration of Chinese character meanings by having participants report whether two individual characters are related in meaning while undergoing fMRI scanning. Pairs of semantically related characters evoked stronger activation than semantically unrelated ones in the left IPL, anterior cingulate cortex, and posterior cingulate cortex, suggesting stronger integration of highly related semantic features. Therefore, we speculate that the left IPL may support recognition of Chinese multi-character words by accessing and/or integrating semantic information during Chinese word reading.

In comparison with activation of the left IPL, activation of the right IPL was less frequently reported in studies of word reading. However, some recent studies found that activation in the right IPL during word reading was specifically modulated by the learning experience of written Chinese. Cao et al. [53] trained two groups of English-speaking adults: one group to learn Chinese characters and the other to study pinyin (an alphabetic system that indicates pronunciation of a character). During the lexical decision task, the character-learning group showed higher activity in the right IPL than the pinyin-learning group. Similarly, Wu et al. [54] found that literate Chinese-speaking adults showed higher activity in the bIPLs than illiterate participants during matching of Chinese characters. These findings are different from those of a previous study on Portuguese-speaking subjects, in which the literate group showed higher activity in the left IPL but lower activity in the right IPL than the illiterate group [55], and suggest that the right IPL may have a unique role in written Chinese processing. The present study provides novel supportive evidence for involvement of the right IPL in the reading of Chinese words. However, cross-language consistency of the role of this region in word reading is unknown and should be investigated in future studies.

Notably, the bIPLs, especially the anterior parts (bilateral supramarginal gyri), are also thought to play a critical role in phonological processing [3, 19, 51, 56]. However, Zhan et al. found that IPL activity during recognition of Chinese multi-character words can hardly be explained as pure phonological processing [21]: although both mixed and pure pseudohomophones sound the same as real words, only the mixed pseudohomophones evoked stronger IPL activation than regular nonwords. These findings, together with evidence in support of the involvement of the IPL in semantic processing [47], collectively indicate that IPL activity during recognition of Chinese multi-character words is more likely to reflect semantic processing rather than phonological processing.

In addition to bIPLs, the anterior and posterior parts of the cingulate cortex were also activated during processing of words and transposable nonwords. These regions have been identified as part of the lexical semantic network [10]. However, their functions remain to be elucidated. Previous research has proposed that the anterior cingulate cortex may process emotional attributes of concepts, and the posterior cingulate cortex may function as an interface between the semantic and episodic memory systems [10]. Alternatively, as introduced above, coactivation of these regions with the left IPL during semantic integration of character meanings has been observed [47]. These findings suggest that the anterior and posterior cingulate cortices may collaborate with IPLs to form a coherent network underlying the semantic integration of character meanings.

In conclusion, the functional anatomy of recognition of Chinese multi-character words was explored in the current study by examining the brain activation associated with the effects of transposable nonwords, lexicality, and word frequency. A conjunction analysis on contrasts for the first two effects eliminated the confounding effect of RT and demonstrated stronger activation for both transposable nonwords and words in the bIPLs and anterior and posterior cingulate cortices. ROI analyses further revealed positive word-frequency effects in the bIPLs and posterior cingulate cortex. These findings have provided evidence supporting the importance of bIPLs and cingulate cortex in recognition of Chinese multi-character words and indicated their involvement in accessing and/or integrating semantic information during Chinese word reading.
